# Update on the similarities between SADS, SIDS, and SUDEP: Three sides of the same pyramid?

**DOI:** 10.1007/s00414-025-03612-0

**Published:** 2025-10-09

**Authors:** Rosario Barranco, Isabella Caristo, Andrea Molinelli, Gabriele Rocca, Nicolò Vernazza, Francesco Ventura

**Affiliations:** 1 Department of Legal and Forensic Medicine, Health Science Department (DISSAL), University of Genova, via De Toni 12, Genova, 16132 Italy; 2 IRCCS-Ospedale Policlinico San Martino Teaching Hospital, Largo Rosanna Benzi 10, Genova, 16132 Italy

**Keywords:** SUDEP, SIDS, SADS, Molecular autopsy, Pathophysiological overlaps, Forensic pathology

## Abstract

Sudden Arrhythmic Death Syndrome (SADS), Sudden Unexpected Death In Epilepsy (SUDEP) and Sudden Infant Death Syndrome (SIDS) present some overlaps and similarities in terms of molecular autopsy and pathophysiological mechanism. Genes associated with cardiac arrhythmias represent promising biomarkers, in light of the growing evidence of neurocardiac interconnections and phenotypic similarities between SUDEP, SADS and also SIDS. The interactions between these different forms of sudden death are reciprocal and could help to understand the mechanism of death that in some cases remains unexplained. In this review of the literature we analyse the overlaps and the common aspects between these different conditions of sudden death, we discuss the clinical, social and medico-legal implications. SIDS, SADS and SUDEP still represent a huge challenge for the Forensic Pathologist and their diagnostic interpretation contains some ambiguities and evaluation difficulties. All cases of sudden death require a thorough cardio-pathological and neuropathological evaluation. A thorough anamnesis and molecular analysis of the major channel protein genes (such as SCN5A, RYR2, KCHN2, and KCNQ1) should be performed in these cases, in addition to histological and toxicological analyses. Only a thorough and multidisciplinary evaluation can help to better define the cases of sudden death, avoid improper classifications and clarify the pathological mechanism more precisely.

## Introduction

Sudden Death (SD) is a devastating event, defined as a fatal outcome occurring within one hour of symptom onset in an individual who appears healthy or whose underlying medical condition does not typically lead to sudden death [1, 2].

In forensic pathology, Sudden Arrhythmic Death Syndrome (SADS) encompasses cases of SD that remain unexplained after comprehensive pathological and toxicological examinations. In the United States, SADS accounts for approximately 35% of deaths in young adult males aged 18–35 years [1]. Long QT syndrome, short QT syndrome, Brugada syndrome, and Catecholaminergic Polymorphic Ventricular Tachycardia (CPVT) are among the most common conditions associated with SADS [3].

Sudden Unexpected Death In Epilepsy (SUDEP) is another condition that can lead to sudden death [4].

The latter is defined as an unexpected and sudden, non-traumatic death in individuals with epilepsy, occurring without a witnessed seizure and without an identifiable cause of death following a thorough investigation, including autopsy and toxicology testing [5, 6]. The presence of comorbidities in addition to epilepsy characterizes SUDEP plus.

Probable SUDEP refers to cases where an autopsy is performed, while possible SUDEP implies the coexistence of epilepsy and another potential cause of death. Near-SUDEP describes a situation where an individual survives a life-threatening epileptic event for at least one hour, with or without comorbidities.

The genetic risk factors for SUDEP remain poorly understood but may involve various mechanisms, including cardiorespiratory dysfunction and autonomic alterations [7–10]. Studies in both animal models and humans have suggested that genetic variants associated with SADS may contribute to some cases of SUDEP [9, 11]. Channelopathies, which affect ion channel function, are implicated in both arrhythmic and convulsive disorders, further highlighting the potential link between these conditions [12–14].

Channelopathies are disorders caused by defects in ion channels and alterations in these structures are implicated in a broad spectrum of diseases. Ion channels are transmembrane proteins that mediate the passive flow of ions into and out of cells, following their electrochemical gradients. This ion movement generates electrical currents that contribute to the establishment and maintenance of the membrane potential [15]. A prominent example is the *SCN5A* gene, which encodes the alpha subunit of the main cardiac sodium channel Nav1.5. This channel is essential for maintaining the normal inward sodium current [16]. Another gene implicated in cardiac abnormalities is *KCNQ1*. In cardiac myocytes, the *KCNQ1* subunit assembles with the KCNE1 subunit (minK) to form a channel complex that generates the delayed rectifier potassium current (IKs), which contributes to the termination of the cardiac action potential [17]. The *KCNH2* gene also encodes a potassium channel, specifically the rapid component of the delayed inward-rectifying, voltage-gated cardiac potassium channel KV11.1. This channel mediates a major repolarizing current in cardiomyocytes [18].The *RYR2* gene encodes ryanodine receptor 2, a key player in intracellular calcium homeostasis and excitation–contraction (EC) coupling. The RyR2 protein is thought to physically interact with the dihydropyridine (DHP) receptor located in the T-tubules. In myocardial cells, depolarization of the plasma membrane activates the DHP receptor, triggering an influx of Ca²⁺. In response, the RyR2 receptor releases calcium from the sarcoplasmic reticulum into the cytosol [19].

Channelopathies can manifest as both arrhythmic and convulsive disorders, suggesting a genetic predisposition to SUDEP [12]. Approximately 14% of refractory epilepsies are associated with electrocardiographic abnormalities [13, 14], and several studies have reported cases of epilepsy in individuals with Brugada syndrome [20]. In many of these cases, the autopsy frequently reveals a complete absence of pathological findings, leading to their classification as “autopsy-negative sudden unexplained deaths” [21]. Consequently, molecular autopsy and a thorough understanding of this subject are paramount.

In infants under one year of age, sudden unexpected death, often referred to as Sudden Infant Death Syndrome (SIDS), remains a significant public health concern [22, 23]. SIDS is defined as the sudden unexpected death of an infant < 1 year of age, with onset of the fatal episode apparently occurring during sleep, that remains unexplained after a thorough investigation, including performance of a complete autopsy and review of the circumstances of death and the clinical history [24].

The triple risk model proposes that SIDS occurs when a vulnerable infant is exposed to adverse environmental factors during a critical developmental period [25, 26]. While rare, cardiac arrhythmias may also contribute to sudden infant death [25, 27, 28].

SADS, SUDEP, and SIDS are diagnoses of exclusion, often relying on circumstantial evidence and negative autopsy findings [29]. Molecular autopsy, which involves the analysis of genetic and molecular markers, can be a valuable tool in elucidating the underlying causes of these conditions.

Emerging evidence suggests that SADS, SUDEP, and SIDS share common molecular and pathophysiological mechanisms, particularly involving cardiac arrhythmias. Genetic factors, such as those associated with ion channel disorders, may increase susceptibility to these conditions [30–33]. The interplay between cardiac and neurological factors is a key area of ongoing research, with the potential to improve our understanding of these devastating events.

This review aims to explore the overlapping features and common aspects of SADS, SUDEP, and SIDS, as well as to discuss their clinical, social, and medico-legal implications.

## Materials and methods

This review included a first phase in which the most cited articles regarding the most common lesion scoring systems were selected. The PRISMA checklist is provided as Supplementary Material Table.

A comprehensive search of the bibliographic databases PubMed, Embase, Web of Science Clarivate from October 30, 2024 to November 3, 2024.

The following terms (including synonyms and closely related words) were used and combined as index terms or free text words. The search was performed with the following wording:

(“SADS” OR “sudden arrhythmic death syndrome” OR “Channelopathy”) AND (“sudden infant death syndrome” OR “SIDS”);

(“SADS” OR “sudden arrhythmic death syndrome” OR “Channelopathy”) AND (“SUDEP” or “epilepsy death”);

(“sudden infant death syndrome” OR “SIDS”) AND (“SUDEP” or “epilepsy death”);

(“sudden infant death syndrome” OR “SIDS” OR “SUDEP” OR “epilepsy death” OR “SADS” OR “sudden arrhythmic death syndrome” OR “Channelopathy”) AND (“molecular autopsy” OR “genetics” OR “pathophysiology” OR “congenital”).

Duplicate articles were excluded. To ensure maximum completeness and accuracy of the search, both controlled terms (MeSH in PubMed, Emtree in Embase) and free text terms were used. In this way, a broad spectrum of relevant studies was identified.

The search results were independently assessed for eligibility. Moreover, references from the retrieved original articles were searched for missing studies. Three researchers conducted independent searches. The research has been conducted over a 30-year period, commencing in 1994.

The publications finally selected for analysis had to meet the following inclusion criteria:

- Focus: Studies on the genetics, pathophysiology, and histopathology of Sudden Arrhythmic Death Syndrome (SADS), Sudden Unexplained Death in Epilepsy (SUDEP), and Sudden Infant Death Syndrome (SIDS). Clinical and forensic studies comparing SADS, SUDEP, and SIDS.
- Language: Articles published in English.
- Publication date: Articles published between 1994 and 2024.
- Study type: Original research articles, case series, literature reviews, and systematic reviews were included.

## Results

The Literature search yielded over 2900 articles. After removing duplicates and irrelevant articles, 197 articles remained eligible for analysis. Article selection was performed through a multi-step process. Initially, titles and abstracts were screened to identify potentially relevant articles, resulting in 133 articles remaining for full-text screening. Subsequently, the selected articles were subjected to a more thorough full-text assessment to verify their actual relevance to the research objectives. 41 articles after a thorough reading and analysis were excluded as off-topic/and did not meet the selection criteria. After a comprehensive review of the full texts, 92 articles were deemed eligible for inclusion in the study.

For several years, genetic and pathophysiological correlations between SADS, SUDEP and SIDS have been proposed.

Long QT syndrome is associated with mutations in at least 12 genes. The most common forms, LQT1, LQT2 and LQT3, are caused by mutations in the KCNQ1, KCNH2 and SCN5A genes, respectively [28]. Although less frequent, mutations have been identified in other genes, including KNCE1, KNCE2 and proteins such as the mink beta subunit of KCNQ1, the HERG beta subunit MiRP1, the potassium channel Kir2.1, the L-type calcium channel – beta4 subunit, caveolin-3, the beta4 subunit of the sodium channel, the Yotiao protein encoded by AKAP9, α1-syntrophin [1, 34–41]. Brugada syndrome is often related to the mutation of the SCN5A gene encoding sodium channels [34, 42]. Mutations in the GPD1L (glycerol-3-phosphate dehydrogenase-1–like) gene and in the CACNA1C and CACNB2b (alpha and beta subunits of the L-type calcium channels) genes have been associated with Brugada syndrome [34, 43, 44]. CPVT is generally an autosomal dominant disease and is associated with mutations in the RYR2 (ryanodine receptor/calcium release channel) gene and the recessive form is associated with mutations in CASQ2 (calsequestrin) [34].

One of the first overlaps between SADS and SIDS was reported by Schwartz et al. [44], in which a SIDS case presented the KCNQ1, P117L mutation (the same as LQT1).

Mutations in KCNQ1, KCNH2/HERG, SCN5A, KCNE2, CAV3, SCN4B, SNTA1 have been associated with SIDS and are also involved in the pathogenesis of LQTS [33, 45–48].

Rare variants of KCNQ1 have been described in 2.5% of a cohort of SIDS cases but this percentage decreased to 1% when pathogenic variants were considered [49, 50].

Arckernam et al. [51] conducted a post-mortem genetic analysis on a large group of SIDS cases, focusing on the SCN5A gene, responsible for the cardiac sodium channel Nav1.5. Specifically, they identified two genetic alterations, A997S and R1826H, in two of the 58 SIDS cases examined.

In addition, the same research group identified other genetic mutations associated with Long QT Syndrome (LQTS) in 3 of 58 (5.2%, 2 SCN5A and 1 KCNH2) white infants and 1 of 34 (2.9%, 1 KCNQ1) black infants [51]. Other cases included a white infant with a G294V-KCNH2 mutation and an African American infant with five channel variants, including T600M-KCNQ1 and V14I-KCNE2 [34, 52]. Arnestad et al. [45] examined seven LQTS susceptibility genes in 201 SIDS cases and found functionally significant rare genetic variants in 9.5% of cases.

The majority of mutations were found in the KCNQ1, KCNH2 and SCN5A genes. Similar percentages have also been reported in Japanese and French studies [53, 54]. A previous research on a French case of Sudden Unexplained Death In An Infant (SUDI) identified a specific genetic variation, called SCN5A c.2923c > t [54].

Cronk et al. [55] in SIDS cases in an African-American population have traced the mutation in the CAV3 gene encoding caveolin-3. In addition, a small percentage of SIDS cases present mutations in the GPD1L gene [56].

A 2007 study analysed the prevalence of RyR2 mutations (in about 2%) in a cohort of SIDS cases and two distinct and novel RyR2 mutations were identified. A S4565R mutation in a 4-month-old white female infant and a R2267H missense mutation in a 6-month-old black female infant [57]. A further study in 2012 describes alterations in RYR2 in 9% of SIDS cases [58].

According to a study by Wang et al. [46] of 274 cases of sudden unexplained death in neonates (141 cases) and non-neonates (133 cases), LQTS-associated mutations were detected in 13.4% of infant cases, with the SCN5A gene representing the most common genetic alteration; however, SIDS-related mutations were also detected in the KCNQ1, KCNH2, KCNE2 genes.

Cheng et al. [59] found a mutation in the SNTA1 gene (associated with a QTLS) in 8 cases of SIDS.

Hertz et al. [60] identified potentially functional genetic variants (including KCNE4 and RYR2) in over a third of sudden unexpected infant deaths. However, only one novel variant in the RYR2 gene was potentially informative according to even more stringent criteria.

Tester et al. [25] found a 12.6% frequency of ultra-rare potentially pathogenic genetic variants in genes associated with genetic heart disease in a cohort of SIDS cases. In particular, ultra-rare non-synonymous variants were observed in European SIDS cases compared to controls when combining the 4 major cardiac channelopathy genes (SCN5A, KCNQ1, KCNH2 and RYR2). Therefore, according to the Authors, channelopathies represent the underlying pathogenic basis for some SIDS cases and this justifies genetic analysis in these cases.

A Dutch study [61] performed on over one hundred SIDS cases evaluated a comprehensive exon screen for cardiac arrhythmia genes (SCN5A, KCNQ1, KCNH2, KCNE1, KCNE2, CACNA1C, CAV3, ANK2 and KCNJ2). A total of 40 DNA variants were identified in 8 cardiac arrhythmia genes in 58.8% of cases. 32.5% (numerically 13) of the DNA variants identified in 6 cardiac arrhythmia genes were significantly associated with SIDS and were observed in 14.7% of SIDS cases. A total of 16 DNA variants were considered likely pathogenic for cardiac arrhythmias. Furthermore, in this study the Authors identified four DNA variants (CACNA1C c.5617 g > a, CACNA1C c.5222c > t, ANK2 c.2249a > g and ANK2 c.5609a > g) significantly associated with SIDS.

In a study [26] performed on 31 SIDS cases, 20% of the cases presented variants of uncertain significance related to cardiac disease–associated genes and in 6% the variants were considered potentially informative (SCN5A Arg1316Gln and KCNJ2 Glu16Gly).

In a very recent study by Cazzato et al. [28] pathogenic variants were found in SLC22A5, SCN5A, MYL3and TTN genes in 4 cases (5.3%) of SIDS, while 99 variants were classified as of uncertain significance (VUS).

Overall, approximately 10–15% of SIDS cases have genetic mutations that overlap with SADS [3, 25, 34, 46, 48, 62–66] and according to further studies [49] approximately 28% of SIDS post-mortem cases have variants in genes encoding cardiac ion channels.

SUDEP, SADS and SADS could also be linked by a common mechanism at the molecular level, namely alterations in ion channels. Common genetic abnormalities suggest a possible shared genetic basis and a complex interaction between genetic and environmental factors [67–72].

Several genes encoding channel proteins are expressed in both the brain and the heart [1, 3] and several studies have shown a molecular overlap between sudden unexpected death in epilepsy and channelopathies [12, 13, 73–77]. Genes such as SCN5A, KCNQ1, KCNH2, RYR2 are associated with both channelopathies and epilepsy and almost 40% of people with LQT2 and 20% of those with LQT1 present an epileptic phenotype [12, 14, 75].

In addition, in 28% of cases with LQTS a seizure phenotype associated with KCNH2, KCNQ1, and SCN5A mutations has been found [78, 79].

One of the major associations between SADS and SUDEP was the SCN5A mutation, expressed in the brain in the limbic system [80].

Several studies suggest that arrhythmogenic variants may contribute to the pathophysiology of SUDEP, in addition to being closely associated with Brugada syndrome and long QT syndrome [13, 81–84]. This could demonstrate the existence of interactions responsible for genetic susceptibility to brain and cardiac dysfunctions [82].

A SCN5A variant (R1623Q) was found in cases of a double phenotype characterized by seizures and QTLS [78].

Parisi et al. [83] described the case of a family with mutation of the SCN5A gene (c.3284G > A p.W1095X) and co-expression of Brugada syndrome and epilepsy. Leong et al. [85] reported the case of a person with SCN5A mutation with temporal epilepsy and Brugada syndrome.

Heron et al. [86] reported the case of a patient with variants in KCNE2 [c.170 T > C (p.I57T)] and SCN5A [c.4868G > A (p.R1623Q)] and electrocardiographic alterations from LQTS and seizure. This channelopathy can be associated with a spectrum of disorders, including epilepsy, cardiac conduction abnormalities, and neuronal dysfunction [87].

According to the Literature, in about 75% of cases of overlap between SADS and SUDEP, the SCN5A, KCHN2, and KCNQ1 genes were involved [7, 12].

In this sense, the KCNQ1 gene that codes for the potassium channel protein is associated with epilepsy and long QT syndrome [20, 88]. Animal studies have highlighted an altered function of channel proteins with a cardio-cerebral phenotype (such as for example KCNQ1 mutation that codes for the Kv7.1 protein) [13, 77].

A reduction in the function of a KCNH2 variant has been statistically associated with SUDEP cases compared to controls [11, 70, 81, 82].

A previous post-mortem genetic analysis on 48 SUDEP cases found some non-synonymous variants in KCNH2 and SCN5A (Arg176Trp and Arg1047Leu in KCNH2, Ala572Asp, Pro1090Leu and Pro2006Ala in SCN5A) of which two (SCN5A Pro1090Leu and KCNH2 Arg176Trp) were already found in SQTL and were directly involved in the mechanism of death [89].

The study by Bagnall et al. [75] analysed cardiac arrhythmia, respiratory control and epilepsy genes by exome sequencing in 61 SUDEP cases. Mutations in common cardiac arrhythmia disease (LQTS) genes were identified in four SUDEP cases (7%) while candidate pathogenic variants in dominant cardiac arrhythmia genes were found in nine cases (15%). The authors identified a de novo SCN5A Ile397Val variant, a nonsense variant Gly924Ala and Arg744 in KCNH2 and a nonsense variant Tyr662 in KCNQ1. These variants had been reported in QLTS and Brugada Syndrome [90, 91].

A case report from several years ago described the presence of a SCN5A R523C mutation in an epileptic woman who died unexpectedly during sleep [74].

Indeed, genetic analyses conducted in SUDEP cases have revealed the presence of potentially dangerous genetic alterations in genes involved in the control of cardiac rhythm (such as KCNQ1) and in genes associated with severe forms of epilepsy (such as SCN1A and DEPDC5) [9, 75, 89, 91, 92].

For example, Zarroli et al. [93] and Omichi et al. [94] report variants in KCNH2 in patients affected by LQTS and seizures.

In a WES, pathogenic variants of the LQTS genes (SCN5A, KCNH2, KCNQ1) were found in 7% of SUDEP cases and variants associated with predisposition to malignant arrhythmias (ANK2, AKAP9, HCN4) were found in 15% [7, 75]. Further studies have highlighted the association between KCNH2 and KCNH2 mutations and SUDEP [95].

KCNH2 variants were found in 11.1% of SUDEP cases compared to 6% of epileptic controls in a recent study performed by Soh et al. [11].

Friedman et al. [9] examined Whole Exome Sequences (WES) derived from brain samples in eight epileptic patients deceased from SUDEP and identified variants in cardiac arrhythmia-associated genes (KCNMB1, KCNIP1, DPP6, JUP, F2, and TUBA3D) that were not present in living epileptic controls.

Overall, KCNH2 alterations are reported in 3% of SUDEP cases and 25–30% of LQTS [87].

A 2016 study [91] performed next-generation sequencing on a cohort of 14 SUDEP cases. Four cases harbored rare variants with complete segregation in genes including SCN1A, FBN1, HCN1, SCN4A, and EFHC1. One case demonstrated a rare variant in KCNQ1 with incomplete penetrance and other cases showed rare variants in the CACNA1A, SCN11A, SCN10A and KCNQ1 genes.

A further study [96] highlighted non-synonymous heterozygous missense pathogenic in exon 6 of the KCNQ1 [variant p.L273F (c.817 C > T)] in a family affected by epilepsy and LQTS.

A Japanese study [97] on 9 cases of SUDEP performed next-generation sequencing to examine 73 genes related to inherited heart disease: the Authors found KCNE1 as pathogenic variants (together with KCNE1) and also 3 other potentially pathogenic variants (MYH6, DSP, DSG2) according to in silico analysis.

In a prospective study the p.D85N SNV mutation in the KCNE1 gene was described in a patient with epilepsy and LQTS [98].

Further studies have detected a pathogenic variant SCN1A (coding for sodium channel protein Nav1.1) in the majority of cases (over 80%) of Dravat syndrome [99–101].

Arrhythmias and seizures were observed in 12 cases of CPVT affected by RYR2 gene mutation [102]. The mutation c.14,806 G > A, p. G4936R of the same gene [103] was found in a case of SUDEP in an 8-year-old child.

Overall pathogenic variants associated with SADS (with main reference to LQTS) were highlighted in about 10–15% of SUDEP cases [7, 81].

Furthermore, autopsy analyses have shown myocardial alterations in SUDEP cases, such as interstitial and perivascular fibrosis, absent in control cases [104]. It has been postulated that sympathetic overactivation can cause myocardial damage in epileptic subjects with consequent eccentric hypertrophy and systolic and diastolic dysfunction [13, 105].

According to an acquired pathophysiological hypothesis, cardiac remodelling and modulation of transcription factors and ion channel expression with subsequent acquired channelopathy can be triggered by over-stimulation of adrenergic receptors, as in chronic epilepsy [13].

From a genetic point of view, genetic overlaps have also been detected between SUDEP and SIDS involving the SCN1A gene [106, 107]. Alterations of the PHOX2B gene have been associated with sudden infant death [108, 109] and have also been correlated to SUDEP [78].

In this context, interesting pathophysiological associations involving the serotoninergic system and respiratory function have been found [9, 110]. Both SUDEP and SIDS appear to be related to a problem in the control mechanism in response to increased carbon dioxide (CO2) in the blood during sleep [101, 107]. Several studies have shown that serotonin in the dorsal raphe nucleus regulates the arousal mechanism [101, 106, 111, 112] with dysregulation of the 5-HT system in SIDS [113]. In both SUDEP and SIDS, dysfunction of arousal, chemoreceptors and brainstem serotonin transmission appear to be involved in causing sudden death [101, 107, 113]. In fact, dentate gyrus abnormalities and 5-HT defects are reported in approximately 40% of SIDS cases [114]. On the other hand, anomalies of the hippocampus (with bilamination of the dentate gyrus) and temporal lobe have been identified in many cases of SIDS [107] as well as in SUDEP [113].

Furthermore, another similarity also concerns the position of the body, as in both cases the corpse is often found in a prone position [107]. Furthermore, several case reports [107, 113] describe epileptic seizures observed in newborns who died soon after.

It is not known whether these SIDS cases have the same anomalies, both in the hippocampus and in the brainstem. It could be that there are two types of sudden infant death, one with problems in the hippocampus and the other with problems in the brainstem. A stress, such as difficulty breathing or a high fever, causes a seizure in these cells. However, there is no protection from these seizures because the areas involved in the control of breathing and heartbeat also do not function well [114].

According to some authors, these anatomical markers of vulnerability to sudden death could also unify the forensic approach to the investigation of SIDS, and SUDEP [114].

This observation raises a question: how many deaths attributed to SIDS could actually be caused by SUDEP, perhaps triggered by a first epileptic seizure, even undiagnosed, in the newborn?

## Discussion

This review aims to summarize the common features described in the literature regarding the pathophysiological mechanism of SIDS, SADS and SUDEP. The latter can be considered heterogeneous states that overlap in some cases. Often in these cases toxicology, autopsy and histology are normal.

From the anatomopathological and pathophysiological point of view, anomalies of the limbic system, hippocampus and serotoninergic system have been proposed to explain both cases of sudden infant death and sudden unexpected death in epilepsy.

From the molecular point of view, variants of the genes encoding the sodium and potassium channel proteins such as SCN5A, KCHN2, and KCNQ1 are those most frequently discovered in cases of SIDS and SUDEP.

Furthermore, the cardiac remodelling theory also appears very interesting: in cases of SUDEP the channelopathy can be acquired and arise following an anomalous output to the heart during chronic epilepsy, determining a proarrhythmic state [13].

Sudden death is a catastrophic event with a huge social impact and on the victim’s family. SIDS, SADS and SUDEP still represent a huge challenge for the Forensic Pathologist and their diagnostic interpretation contains some ambiguities and evaluation difficulties [1, 115].

In particular, SIDS and SUDEP essentially represent diagnoses of exclusion. Sudden infant death syndrome represents a non-diagnosis, or the admission of not having found the cause despite having performed all postmortem tests (autopsy, histology, toxicology, microbiology, genetics). Basically, it is a construct to elegantly say: “I don’t know”. SUDEP provides a classification that is starting to be ambiguous and outdated [1]. In these cases, the risk is to be misled by the circumstantial data of a sudden death in an epileptic person.

The definition of SUDEP itself seems a bit outdated: it refers to “sudden, unexpected death, witnessed or not witnessed […] in which the post-mortem examination does not reveal a toxicological or anatomical cause to which the death can be attributed”. Some causes of sudden death can be difficult to identify and in these cases the autopsy as well as the histological examination may be normal and only in-depth genetic analyses can suggest the cause of death. However, not all Forensic Medicine centres have the possibility of performing in-depth genetic investigations. Consequently, there is a potential for overdiagnosis of SUDEP in cases where the sole evidence is a history of epilepsy, particularly when traditional autopsy findings are inconclusive. This is the case of channelopathies/arrhythmias that can contribute to the death of these people as a primary electrical event [1]. In fact, the results of this review highlight how many genetic mutations of the channel proteins causative of SADS are present in SUDEP. More than 10–15% of SUDEP cases present pathogenic variants associated with SADS (with main reference to LQTS [7, 81]. Similarly, cardiac channelopathies would represent the underlying cause of a portion of SIDS, also in this case in about 10–15%. On the other hand, the neurological pathophysiological evidences common between SIDS and SUDEP represent an interesting source of scientific debate.

In some cases, the etiological overlap between these different definitions of sudden death may recognize a single common cause. In this sense, a part of SIDS may have a pathological basis in channelopathies, another part of SIDS in a common cause with SUDEP. The only difference would be the onset of the fatal event: in some cases in the neonatal period, in others in adulthood. At the same time, a part of SUDEP cases would actually be due to a cardiac channelopathy.

Scientific studies have verified the role of variants of the genes for ion channel proteins in epilepsy and arrhythmia, leading to the theory of “cardio-cerebral channelopathy” [87].

In fact, according to some authors [20] genetic mutations related to channelopathies (specifically SCN5A) have a phenotypic expression dependent on age, with cerebral characteristics that occur earlier in Life and cardiac manifestations that appear later. A further study suggests that patients with epilepsy experience a 12-year delay in the diagnosis of channelopathy [20, 116].

Therefore, autonomic dysfunction and structural cardiac disease may be the candidate key to understanding the pathophysiology [13].

The pertinent question is whether questionable genetic variants and structural abnormalities also contribute to the increased risk of sudden death.

For example, in cases of SUDEP with variants of channelopathy genes, some authors have proposed a two-phase model: the first is the pre-existing individual vulnerability on which cardiac stress is inserted that triggers the fatal arrhythmic event.

One of the major problems in these cases (SADS, SIDS and SUDEP) is misinterpretation: several cases of channelopathy misdiagnosed as primary epilepsy and vice versa have been reported [87, 117] and this problem also concerns the forensic field.

The scientific community needs to be careful not to overstate these “overlaps”: if on the one hand some cases can recognize a single common cause, in others some similarities can only be coincidental and have no common role in the cause of death. For example, some variants of uncertain significance in channel protein genes in cases of SIDS or SUDEP have no value or contribution with respect to the mechanism of death and can only represent confounding factors. A high proportion of variants in genes for ion channel proteins associated with SIDS constitute a genetic background without a pathogenic role [49].

Therefore, one of the major problems is the correct interpretation of genetic variants: in fact, an adequate classification is necessary to allow specialist advice to family members. Often determining whether a variant alters protein function is very difficult [7]. Incorrectly assigning a non-pathogenic variant can determine other similar avoidable events in family members. Similarly, mistakenly considering a dubious variant as pathogenic can also damage the family [25]. This is the case of the family described by Ackerman et al. [118] in which adverse effects occurred due to a necessary treatment due to a misinterpreted KCNQ1 variant.

Consequently, in case of VUS the physician finds himself in a difficult situation, between a rock and a hard place: a lack of diagnosis can expose the family members to risks while an overdiagnosis can expose the relatives to useless and sometimes even harmful treatments.

The finding of a pathogenic genetic variant has profound consequences for the clinical management of the family. Therefore, the communication of laboratory results should be accurate and accompanied by a careful assessment of the co-segregation of the variant. The importance of this analysis is widely recognized by the international scientific community, as highlighted by the European recommendations [119].

In any case, even now the pathological contribution of variants of uncertain significance remains unknown and further studies are needed to validate conclusions.

Furthermore, the relative frequency of familial versus sporadic mutations in channel protein genes has not been definitively established [34].

Another problem concerns antiepileptic treatment since some drugs (such as lamotrigine) can have an arrhythmogenic action [13]: can this pharmacological side effect precipitate the arrhythmic condition in a person with VUS? That is, can the two effects add up and determine an arrhythmic death? This question is very complicated to answer and there is no in-depth scientific knowledge.

It is extremely difficult whether seizures increase the risk of sudden death in people with pathogenic variants [95]. To fully understand the role of these genetic variants, functional studies are necessary, in order to identify any pathogenic mechanisms that predispose to sudden death during a seizure [91].

Furthermore, a further difficulty concerns the difficulties in evaluating SADS itself and the lack of data with possible misdiagnosis. For example, a seizure can be interpreted as epilepsy instead of cardiac syncope [68]. Classifying deaths in people (both newborns and adults) with a single event similar to an epileptic seizure is extremely difficult [68].

Another issue to consider is the role and availability of molecular autopsy: although it is foreseen in cases of sudden cardiac death and SIDS, its role in SUDEP is not defined [120]. Furthermore, in many Forensic Medicine centres they do not have routine access to genetic tests and the costs could burden families. Barriers related to costs, local knowledge and skills can drastically reduce the use of genetic tests [7].

The risk is that in many cases with non-diagnostic autopsy the necessary genetic investigations are not performed and the cause of death remains undetermined.

The involvement of associations and specialized teams can be fundamental to inform families about the importance and benefits of molecular autopsy also for the health of the closest relatives [7].

The results of genetic tests on family members can indicate which preventive treatments or which drugs to avoid to reduce the risk of similar events. To ensure a comprehensive approach, a multidisciplinary team of experts is needed, including geneticists, pathologists and medical specialists (such as cardiologists, neurologists and paediatricians) [7].

In conclusion, it is evident that the relationship between genotype and clinical phenotype is often not linear [20]. A minority of SUDEP and SIDS cases may have a channelopathy as the cause. Similarly, a portion of SIDS and SUDEP cases may be due to abnormalities of the serotonergic and limbic systems. However, the exact role of these “overlaps” is not always easy to clarify and in a portion of these cases the exact pathological mechanism is not yet ascertainable or is difficult to identify despite scientific efforts.

Certainly all cases of sudden death require a thorough cardio-pathological and neuropathological evaluation. Furthermore, a thorough anamnesis and molecular analysis of the major channel protein genes (such as SCN5A, RYR2, KCHN2, and KCNQ1) should be performed in these cases [33, 67], in addition to histological and toxicological analyses.

Only a thorough and multidisciplinary evaluation can help to better define the cases of sudden death, avoid improper classifications and clarify the pathological mechanism more precisely.
